# CircRNA-Encoded Peptides or Proteins as New Players in Digestive System Neoplasms

**DOI:** 10.3389/fonc.2022.944159

**Published:** 2022-07-22

**Authors:** Enqing Meng, Jie Deng, Rongqi Jiang, Hao Wu

**Affiliations:** Department of Oncology, The First Affiliated Hospital of Nanjing Medical University, Nanjing, China

**Keywords:** digestive cancers, peptides, circular RNAs, translation, signaling pathways

## Abstract

Circular RNAs (circRNAs) were considered non-coding RNAs. Nowadays, a large number of studies have found that these RNAs contain open reading frames that can be translated in a cap-independent manner, such as internal ribosome entry site (IRES) and N6-methyladenosine (m6A). The encoded peptides or proteins affect the occurrence and development of tumors by regulating the Yap-hippo and the Wnt/β-catenin signaling pathways, as well as the malignant progression of tumors through phosphorylation and ubiquitination of specific molecules. This review will summarize the regulation of circRNA translation and the functional roles and underlying mechanisms of circRNA-derived peptides or proteins in digestive tract tumors. Some circRNA-encoded peptides or proteins may be used as tumor biomarkers and prognostic factors for early screening and treatment of clinical gastrointestinal tumors.

## Introduction

Only 1-2% of the human genome encodes proteins, while non-coding RNAs (ncRNAs) account for most of the RNAs, suggesting that ncRNAs represent most of the human transcriptome. Previously, circular RNAs (circRNAs) were a unique subclass of ncRNAs characterized by covalently closed loops, considered “evolutionary junks”. However, accumulating evidence indicated that circRNAs play a critical role in physiological regulation and various diseases (1, 2). Given the lack of typical mRNA characteristics, circRNAs are often considered a subtype of ncRNAs (3). However, some characteristics of circRNAs suggest translational potential. For example, most circRNAs are composed of exon sequences, mainly located in the cytoplasm, and may carry a translatable open reading frame (ORF) containing a start codon (4). A study in 1995 showed that the synthetic circRNA recruits 40S ribosomal subunits and translate through an internal entry site to produce detectable peptides in human cells (5).

Currently, masses of translated circRNAs have been detected with the advent of advanced high-throughput technology, including RNA sequencing (RNA-seq) combined with polysome profiling and circRNA-specific bioinformatics algorithms (6–9). Natural circRNA translation does not depend on the m7G cap but on internal ribosome entry elements, including IRES, such as m6A-IRES (MIRES) (6, 8, 10, 11). Furthermore, circRNAs can be translated through a rolling circle amplification (RCA) mechanism (9, 12). The peptides or proteins encoded by circRNAs can modulate various tumor-related physiopathological processes. For instance, MAPK1-109aa is encoded by circMAPK1, which can compete with MEK1 and inhibit the activation of MAPK signaling pathway and suppress the proliferation and metastasis of GC cells (13). CircDIDO1 induces apoptosis in gastric cancer cells by encoding DIDO1 protein isoform which interacts with the poly ADP-ribose polymerase 1(PARP1) protein to inhibit its DNA repair ability (14). The translation function of circRNAs remarkably enriches genomics and proteomics and provides a new perspective for tumor diagnosis and treatment.

Due to the change in human eating habits, digestive cancers, such as colorectal cancer, gastric cancer, and liver cancer, are common worldwide (15). Therefore, the survival rate of digestive cancer patients can be increased *via* early diagnosis and therapy. In this review, we illustrated how peptides encoded by circRNAs are translated. We assessed a series of studies on the association of peptides or proteins encoded by circRNAs with gastrointestinal tumors to classify the effects of such peptides or proteins on tumors, such as cell proliferation, metastasis and apoptosis.

## Translation of CircRNA-Derived Peptides or Proteins

Protein synthesis in eukaryotes consists of four stages, initiation, elongation, termination, and ribosome recycling (16), wherein the initiation stage is the rate-limiting stage of translation. The classical translation of eukaryotic mRNAs relies on the m7G cap for the recognition of the cap-binding protein initiation factor eIF4E complex, including eIF4E, eIF4G (a scaffold protein), eIF4A (a helicase protein), and the assembly of the 43S initiation complex to direct protein synthesis (17). However, cap-independent translation of circRNAs requires IRES or MIRES to bind to the initiation factor eIF4G2 or eIF3 complex containing eIF4G2, eIF4A, and eIF4B, anchoring the 43S complex for protein translation (18, 19) or requiring infinite ORFs and initiation codons to initiate translation in rolling translation pathways (8, 12, 20). Next, we will assess the three ways of circular RNA translation.

### IRES-Dependent CircRNA Translation

Due to the lack of 5’ cap and 3’ terminal structure, the circRNAs do not have the ability to encode proteins. Recently, this theory has been challenged by the discovery of IRES elements. An IRES-mediated mechanism is one of the widely accepted methods for the translation initiation of circRNAs. IRES, a secondary structure sequence located in the noncoding region of the 5’-end of mRNA, can directly recruit ribosomes to initiate translation (21). Originally, these IRES elements were detected in the sequences of some viral and cellular mRNAs (6). They mediate translation initiation without prior scanning through the 40S subunit from the 5’ end of the IRES-containing mRNA. Thus, 40S subunits enter the IRES-containing mRNAs either by direct binding to the IRES elements or at the 5’ end of the mRNA and then transferring to the IRES (22, 23). Typically, these IRES elements require the IRES-transacting factors (ITAFs) to recruit ribosomes on the internal structure of mRNAs and initiate translation (24).

**Figure 1:**
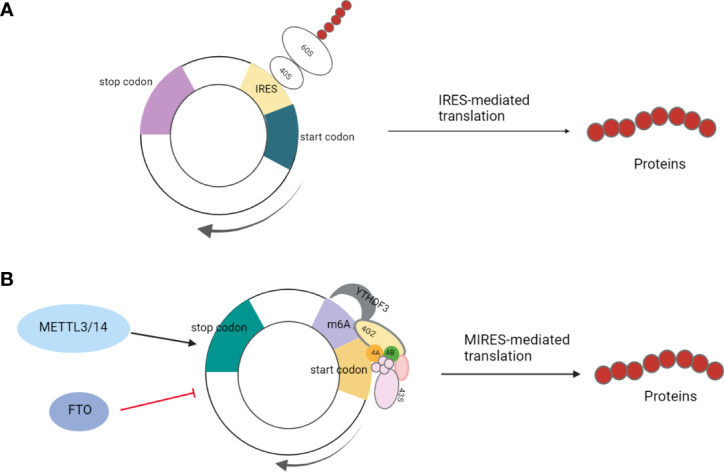
**(A)** IRES-mediated translation: Under physiological and environmental stresses, such as hypoxia, heat shock, or viral infection, eukaryotic mRNA initiates IRES-mediated translation to compensate for defective cap-dependent translation; **(B)** MIRES-mediated translation: In cooperation with the initiation factor eIF4G2 and the m6A reader YTHDF3, a single m6A site is sufficient to initiate circRNA translation. This mode of translation is enhanced by methyltransferase METTL3/14 and inhibited by the demethylase FTO.

A previous study has shown that eukaryotic mRNAs can initially be translated through the IRES-mediated process under physiological and environmental stresses, such as hypoxia, heat shock, or viral infection (**Figure 1A**) (25). This is an alternative translation mechanism in eukaryotes that compensates for defective cap-dependent translation. Some studies have shown that this model is optimal for circRNAs (26, 27). For instance, peptides were encoded by circ-ZNF609 in a cap-independent manner and were initiated by IRES elements in noncoding regions (6). IRES-mediated translation is ubiquitous in circRNAs, and studies have shown that circRNAs can produce the peptides or proteins involved in colon, liver, and gastric cancer development (13, 28–30). For example, Pan et al. verified the activity of IRES in circFNDC3B by dual-luciferase assay, demonstrating that circFNDC3B encodes circFNDC3B-218aa with an IRES-mediated translation initiation mechanism; this protein can inhibit colon cancer progression and epithelial-mesenchymal transition (EMT) (29).

### MIRES-Dependent CircRNA Translation

In addition to regular hairpin-like IRESs, short sequences containing m6A sites can also act as specialized IRESs to initiate the translation of circRNAs. m6A is an abundant modification in mRNA and DNA in higher organisms, with the addition of a methyl group at the N6 position of RNA adenosine (31–33). Yang et al. found that a single m6A site is sufficient to initiate circRNA translation, which also requires initiation factor eIF4G2 and the m6A reader-YTH N6-Methyladenosine RNA Binding Protein 3(YTHDF3). Moreover, this mode of translation is enhanced by the methyltransferase methyltransferase-like 3/14(METTL3/14), suppressed by demethylase fat mass and obesity-associated protein (FTO), and activated under heat shock conditions (11). For example, the reverse splicing of exons 2 and 3 of tumor suppressor gene *ARHGAP35* forms circARHGAP35, which encodes a truncated protein without Rho gap domain composed of four FF domains and is mediated by the starting codon modified by m6A (**Figure 1B**) (10).

### Rolling Circle Amplification (RCA)-Mediated Translation

Several studies have shown that IRES and m6A are crucial common elements for translation initiation of circRNAs, and RCA is a putative mechanism for circRNAs’ translation (21). In 2015, Abe et al. designed a circRNA sequence with unlimited ORF but without any of the above elements necessary for translation initiation to test its translational efficacy in the eukaryotic systems. The results suggested that circRNAs with unlimited ORFs can be translated by RCA (34).

## CircRNA-Encoded Proteins or Peptides in the Digestive System Neoplasm

With social development, environmental changes, and changes in people’s lifestyles, the incidence of digestive system diseases is increasing every year. Due to the fast-paced life and significant work pressure of modern people, most people’s digestive systems are in a sub-healthy state. Compared to the high incidence of gastrointestinal tumors, the early diagnosis rate is low (35). Many gastrointestinal tumors have insidious onset and no symptoms in the early stage. The majority of patients were in the middle and late stages. Several studies reported that the peptides or proteins encoded by circRNAs have an impact on the occurrence and development of digestive tract tumors (12, 29, 36, 37). We have listed circRNAs acting as template for translation in human digestive tract tumors in **Table 1**. Thus, the early screening rate of tumors and the prognosis and survival of patients can be improved by studying the peptides or proteins encoded by these circRNAs as diagnostic markers or drug targets. In the following section, we categorized the effects of circRNA translation products on gastrointestinal tumors.

**Table 1:** Summarsy of peptides encoded by circular RNAs in gastrointestinal tumors.

circRNA	Peptides or proteins	Length (aa)	Expression (Up/Down)	Tumor	Effects	References
circFNDC3B	circFNDC3B218aa	218	Down	Colon cancer	EMT	(29)
circPPP1R12A	circPPP1R12A-73aa	73	Up	Colorectal cancer	Proliferation and metastasis	(13)
hsa_circ_0006401	Hsa_circ_0006401 peptide		Up	Colorectal cancer	Proliferation and metastasis	(36)
cGGNBP2	cGGNBP2-184aa	184	Up	Intrahepatic cholangiocarcinoma	Proliferation and metastasis	(37)
circARHGAP35			Up	Liver cancer	Proliferation and metastasis	(10)
circβ-catenin	β-catenin	370	Up	Liver cancer	Proliferation and metastasis	(38)
CircAXIN1	AXIN1-295aa	295	Up	Gastric cancer	proliferation and metastasis	(39)
circMAPK14	circMAPK14-175aa	175	Down	Colorectal cancer	Proliferation and metastasis	(40)
circMAPK1		109	Down	Gastric cancer	Proliferation and metastasis	(16)
hsa_circ_0061137	DIDO1		Up	Gastric cancer	Apoptosis	(41)
circMRPS35	circMRPS35-168aa	168	Up	Liver cancer	Tumor resistance	(14)

### Effects on EMT

Pan et al. reported that circFNDC3B originates from exons 5 and 6 of *FNDC3B* gene in chr3 and encodes a 218-amino acid (aa) novel protein that was termed the circFNDC3B-218aa. The product of circFNDC3B exerted its tumor-suppressive effect in Snail/FBP1/EMT axis. Fructose-Bisphosphatase 1(FBP1) is one of the limiting enzymes in gluconeogenesis, playing a critical role in glucose metabolism. Notably, the Warburg effect refers to a metabolic shift from glycolysis to aerobic phosphorylation, which affects the development of EMT and promotes tumor malignancy. This phenomenon provides abundant nutrients to cancer cells while initiating extracellular matrix destruction and inducing metastasis. The study found that circFNDC3B-218aa inhibited tumor progression and EMT by alleviating the inhibitory effect of Snail on FBP1 in colon cancer (29).

### Effects on Tumor Proliferation and Metastasis

Zheng et al. provided evidence that circPPP1R12A encoded a conserved small peptide, circPPP1R12A-73aa. The study used nude mouse xenografts for a series of experiments and found that it was circPPP1R12A-73aa, but not circPPP1R12A, that promoted colorectal cancer proliferation and metastasis. Also, the analysis of subclasses of signal transduction pathways in the KEGG pathway database found that circPPP1R12A-73aa affected the hippo-Yap signaling pathway. Next, the colon cancer cell lines overexpressing circPPP1R12A-73aa were treated with a Yes-associated protein 1(Yap1) specific inhibitory peptide 17, which showed that peptide 17 significantly alleviates the promotion of the proliferation, migration, and invasion abilities of colon cancer cells by circPPP1R12A-73aa overexpression (42).

Collagen α-3(VI) chain (COL6A3), encoded by the host gene *COL6A3*, is a major component of the extracellular matrix and structurally has a short triple-helical domain and two large globular N- and C-terminal non-collagen domains (38). Endotrophin is a COL6A3-cleaved C5 domain fragment that directly regulates cancer phenotypes by activating the TGFβ-dependent signaling pathways (39). In accordance with the data from The Cancer GenomeAtlas Program(TCGA), COL6A3 expression levels were higher in colorectal cancer tissues compared to the normal tissues. In the study by Zhang et al., hsa_circ_0006401 results from 2–4 exons of its host gene *COL63* and is localized in the cytoplasm. Hsa_circ_0006401 encodes a novel 198-aa functional microprotein that reduces the mRNA and protein levels of host genes *COL6A3* and TGFβ1 and promotes colorectal cancer proliferation and metastasis by protecting *COL6A3* mRNA from degradation (37). In addition, they speculated that the hsa_circ_0006401 microprotein might be involved in the decay process of poly(A) mRNA as an RNA-binding protein because the poly A tail maintains the activity of the mRNA as a translation template and increases its stability (40). Moreover, the hsa_circ_0006401 peptide is closely related to poly(A) binding and mRNA processing (37).

**Figure 2:**
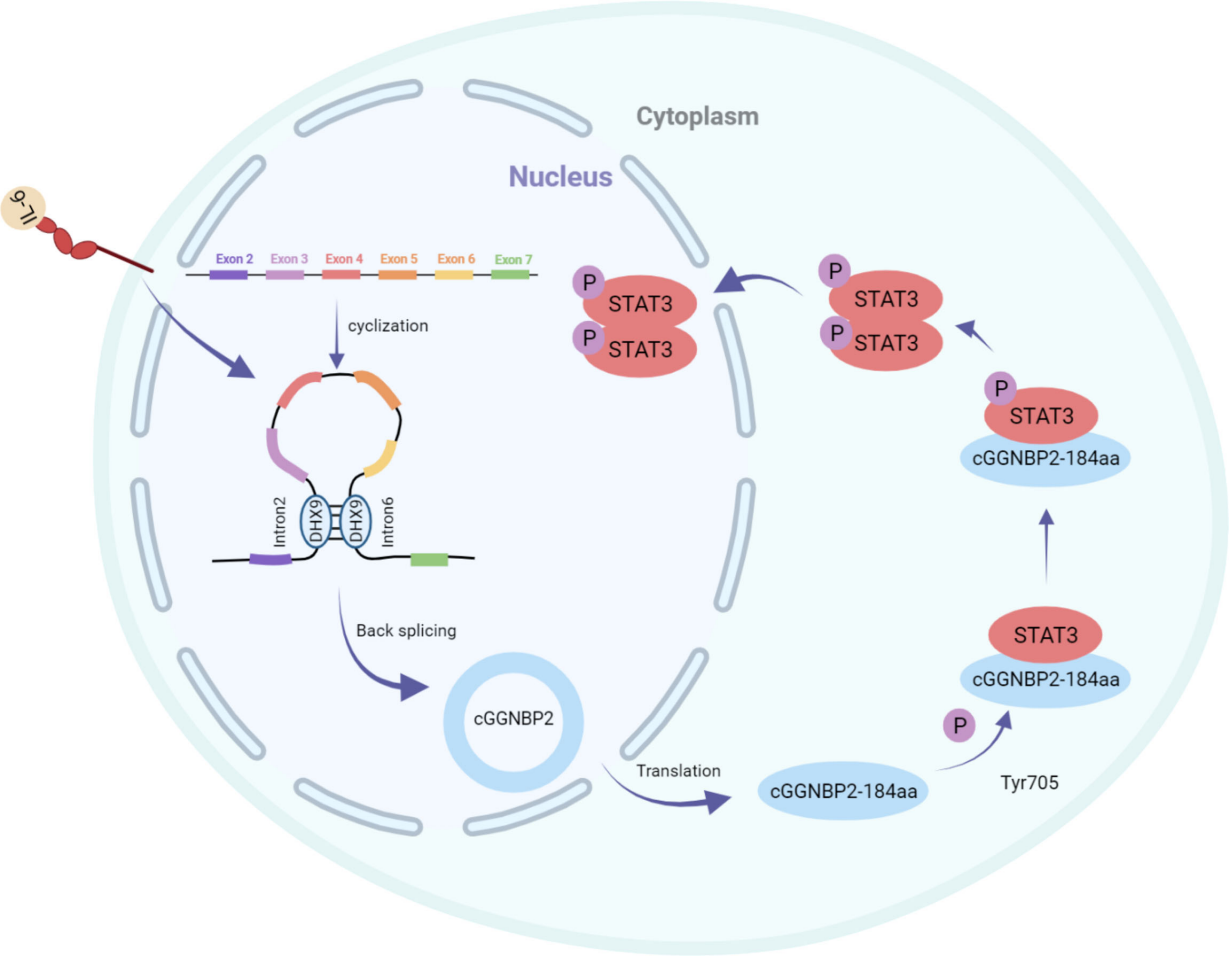
cGGNBP2 is derived from a circRNA of exons 3–6 of gametophyte-binding protein 2, encoding cGGNBP2-184aa. It interacts with signal transducer and activator of transduction 3 (STAT3), enhances its Tyr705 phosphorylation, which is susceptible to the localization to the nucleus and activates the transcription of target genes, thereby promoting the proliferation and metastasis of intrahepatic cholangiocarcinoma cells. IL-6 significantly upregulates the expression level of cGGNBP2 by downregulating the expression of DHX9.

Li et al. identified the protein cGGNBP2-184aa encoded by cGGNBP2, a circRNA derived from exons 3–6 of gametophyte-binding protein 2 by mass spectrometry and Western blotting. This peptide directly interacted with signal transducer and activator of transduction 3 (STAT3) enhancing its phosphorylation at Tyr705, followed by translocation of phosphorylated STAT3 to the nucleus to activate the transcription of the target genes, thereby promoting the proliferation and metastasis of intrahepatic cholangiocarcinoma cells. The translation of cGGNBP2 protein is regulated by IL-6 and DEH-box helicase 9 (DHX9); IL-6 significantly upregulates cGGNBP2 levels by downregulating DHX9 expression (**Figure 2**) (43).

**Figure 3:**
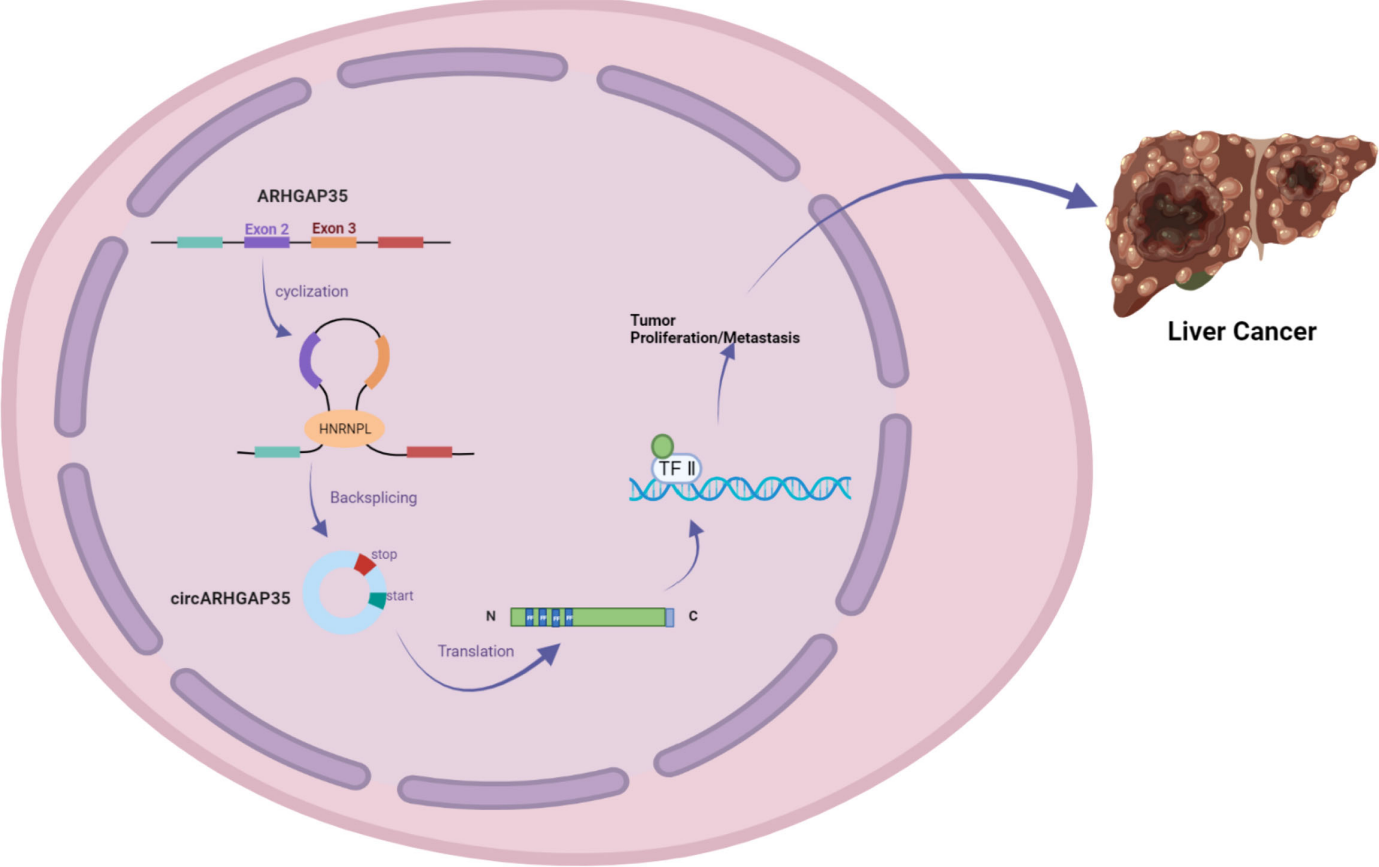
circARHGAP35 is formed by back-splicing of exons 2 and 3 of the tumor suppressor gene ARHGAP35 (also known as P190-a), and the RNA-binding protein HNRNPL promotes circARHGAP35. circARHGAP35 encodes a truncated protein containing four FF domains and lacking the Rho-GAP domain. This truncated protein mainly resides in the nucleus and interacts with the TFII-I protein to promote the proliferation and metastasis of hepatoma cells.

In addition, Li et al. used RNA-seq analysis of ribosomal RNA-depleted total RNA to analyze the circRNA transcripts from 12 paired hepatocellular carcinoma (HCC) and adjacent cancer tissues. qRT-PCR assays identified a circRNA with exons 2 and 3 of the *ARHGAP35* gene (hereafter referred to as circARHGAP35) that was upregulated in HCC. circARHGAP35 contains a large ORF with an m6A-modified start codon in the linker sequence, encoding a truncated protein containing four FF domains and lacking the Rho-GAP domain. The circARHGAP35 protein mainly resides in the nucleus and interacts with TFII-I to promote the proliferation and metastasis of liver cancer cells. HNRNPL was shown to promote the biogenesis of circARHGAP35, and its high expression level upregulates the circARHGAP35 in HCC (**Figure 3**) (10).

**Figure 4:**
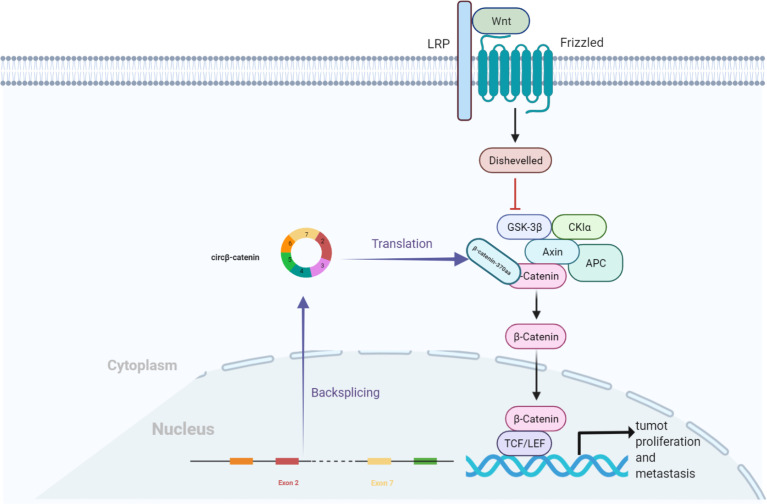
circβ-catenin production of β-catenin isoforms by antagonizing GSK3β, which induces β-catenin phosphorylation and degradation. Nuclear β-catenin interacts with the TCF/LEF complex, which triggers the transcription of target genes and promotes the proliferation of hepatocellular carcinoma cells.

Moreover, Liang et al. found that circβ-catenin was mainly localized in the cytoplasm (41). Compared to the adjacent normal tissues, it was highly expressed in liver cancer tissues, while the knockdown of circβ-catenin inhibited cancer cell growth *in vivo* and *in vitro*. The Wnt/β-catenin pathway is widely involved in multiple pathological events, such as tumor growth, evasion of programmed cell death, cell fate determination, and maintenance of self-renewal capacity (44). circβ-catenin generates a novel 370-aa β-catenin isoform, β-catenin-370aa, which acts as a decoy for GSK3β, preventing it from binding to full-length β-catenin, thereby evading GSK3β-induced β-catenin degradation. The degradation of catenin causes β-catenin accumulation in the cytoplasm, followed by the translocation of β-catenin from the cytoplasm to the nucleus. The nuclear β-catenin interacts with the T-cell factor/lymphoid enhancer-binding factor (TCF/LEF) complex and subsequently initiates the transcription of target genes, thereby promoting liver cell growth and metastasis (**Figure 4**) (41).

In another study, Peng et al. demonstrated that circ AXIN1 consists of exon 2 of the parental gene *AXIN1* that is 959 nt long. CircAXIN1 is highly expressed in GC tissues compared to its expression in paired adjacent normal gastric tissues. The overexpression enhances GC cell proliferation, migration, and invasion, while the knockdown inhibits GC cell malignant behavior *in vitro* and *in vivo*. The study also performed nuclear and cytoplasmic RNA extraction combined with polymerase chain reaction (PCR) and fluorescence *in situ* hybridization (FISH) and found that circAXIN1 was mainly localized in the cytoplasm. CircAXIN1 encodes a novel protein of 295 aa, AXIN1-295aa. The RGS domain of AXIN1-295aa interacts with a 25 aa SAMP from APC, i.e., AXIN1295aa competes with AXIN1 for binding to APC. Consequently, it competes with APC and GSK3β in the destruction complex, preventing AXIN1, CKα, and GSK3β from forming a normal destruction complex. β-catenin then translocates to the nucleus and activates the downstream genes to promote gastric cancer cell proliferation and migration (45).

circMAPK14 (mitogen-activated protein kinase 14) is formed by reverse cleavage of MAPK14 precursor mRNA, and the encoded 175 amino acid polypeptide circMAPK14-175aa can competitively bind to MKK6 to reduce the nuclear translocation of MAPK14. This in turn promotes ubiquitin-mediated degradation of FOXC1, rendering it unable to activate the transcription of downstream molecules, and thus blocking the proliferation and metastasis of colorectal cancer cells. Due to its decreased level, FOXC1 can promote the transcription of U2AF2 and enhance the biogenesis of circMAPK14, forming a positive feedback mechanism to enhance the inhibitory effect of this peptide on the proliferation and metastasis of colorectal cancer (30).

MAPK signaling cascade, with the Ras-Raf-MEK-MAPK signaling pathway, is involved in various cellular and physiological processes essential to life (46). When extracellular stimulators bind to transmembrane receptors, inactive Ras-GDP in the plasma membrane is converted to active Ras-GTP, which then stimulates active homodimerization consisting of A-Raf, B-Raf, and C-Raf formation of dimeric or heterodimers. Raf enzymes catalyze the phosphorylation and activation of bispecific mitogen-activated protein kinase kinases (MEKs); the activated MEKs stimulate MAPK and its downstream factors. Jiang et al. demonstrated that circMAPK1 (hsa_circ_0004872) was downregulated in gastric cancer tissues compared to the adjacent normal tissues, which inhibited the proliferation and metastasis of gastric cancer cells *in vitro* and *in vivo*. It also encoded a new protein with a length of 109 aa, MAPK1-109aa. This peptide competes with MEK1 as a tumor suppressor to reduce MAPK1 phosphorylation, which in turn inhibits the activation of MAPK1 and its downstream factors, thereby inhibiting the proliferation and metastasis of GC cells (13).

### Effect on Apoptosis

Hsa_circ_0061137 is formed by back-splicing of exons 2–6 of the linear transcript of the *DIDO1* gene, 1787 nt in length (henceforth termed as circDIDO1). Bioinformatics analysis by Zhang et al. showed that circDIDO1 has IRES, an ORF, and m6A modification. The study by Zhang et al. found that circDIDO1 encodes a novel DIDO1 protein isoform, which interacts with the poly ADP-ribose polymerase 1 (PARP1) protein to inhibit its DNA repair ability. When single-strand DNA breaks occur in cells, PARP-1 is actively involved in the repair process through various mechanisms. PARP-1 recognizes single-strand DNA breaks through the DNA-binding domain (DBD) and modifies the receptor protein to regulate its conformation, stability, and activity through the CAT domain, thereby repairing DNA damage. PARP-1 inhibition impairs DNA damage repair and induces apoptosis in gastric cancer cells (14).

### Correlation With Tumor Chemoresistance

Li et al. demonstrated that circMRPS35 was highly expressed in HCC post-sorafenib treatment based on the analysis of RNA-seq, indicating that circMRPS35 is associated with chemotherapy (28). circRNADb analysis revealed that circMRPS35 has two IRES putative regions that may encode a 168-aa peptide, which could encode circMRPS35-168aa; this peptide was significantly induced by cisplatin, doxorubicin(DOX), and etoposide. Further experiments demonstrated that overexpressed circMRPS35-168aa mainly induced cisplatin resistance. Western blot results showed that high expression of circMRPS35-168aa counteracted cisplatin-induced high levels of cleaved Caspase-3, an inactive enzyme class that plays a key role in apoptosis (47). These findings demonstrated that circMRPS35 encodes a novel peptide circMRPS35-168aa that is significantly induced by chemotherapeutic drugs and promotes cisplatin resistance in HCC cells (28).

## Conclusions

Peptides or proteins encoded by circRNAs have gained significant attention. Some studies have identified the presence and importance of functional peptides encoded by circRNAs. Herein, we reviewed recent advances in small peptides- or proteins-regulated human digestive tract tumors encoded by circRNAs. This review suggested that future research on functional peptides encoded by circRNAs may be investigated with respect to the following aspects, whether there are other translational mechanisms of circRNAs, the factors and specific mechanisms that affect the protein encoded by the circular RNA, and whether there are other factors that affect the function of the functional peptides encoded by the circRNAs. Based on the current research on circRNA-encoded peptides or proteins in digestive tract tumors, the potential development value and clinical efficacy of circRNA-encoded functional peptides have been discovered. In the future, this approach may be routinely used in cancer research, primary screening, treatment and prognosis.

## Author Contributions

HW designed this study. EM, JD, and RJ cowrote the manuscript. EM prepared the figures. All the authors discussed the results and commented on the manuscript. All the authors have read and approved the final manuscript.

## Funding

This study was supported by a grant from the National Natural Science Foundation of China (No. 81301898) and the Beijing Xisike Clinical Oncology Research Foundation (sy2018-249).

## Conflict of Interest

The authors declare that the research was conducted in the absence of any commercial or financial relationships that could be construed as a potential conflict of interest.

## Publisher’s Note

All claims expressed in this article are solely those of the authors and do not necessarily represent those of their affiliated organizations, or those of the publisher, the editors and the reviewers. Any product that may be evaluated in this article, or claim that may be made by its manufacturer, is not guaranteed or endorsed by the publisher.
